# Free fatty acid receptors beyond fatty acids: A computational journey to explore peptides as possible binders of GPR120

**DOI:** 10.1016/j.crfs.2024.100710

**Published:** 2024-03-04

**Authors:** Lorenzo Pedroni, Florinda Perugino, Fabio Magnaghi, Chiara Dall’Asta, Gianni Galaverna, Luca Dellafiora

**Affiliations:** a Department of Food and Drug, University of Parma, Parma, Italy; b Department of Biology, University of Naples Federico II, Naples, Italy

**Keywords:** Bioactive peptides, GPR120, Molecular modelling, Fatty taste, Virtual screening

## Abstract

Free fatty acids receptors, with members among G protein-coupled receptors (GPCRs), are crucial for biological signaling, including the perception of the so called “fatty taste”. In recent years, GPR120, a protein belonging to the GPCR family, drew attention as an interesting pharmacological target to cope with obesity, satiety and diabetes. Apart from long chain fatty acids, which are GPR120 natural agonists, other synthetic molecules were identified as agonists expanding the chemical space of GPR120's ligands. In this scenario, we unveiled peptides as possible GPR120 binders toward a better understanding of this multifaceted and relevant target. This study analyzed a virtual library collecting 531 441 low-polar hexapeptides, providing mechanistic insights on the GPR120 activation and further extending the possible chemical space of GPR120 agonists. The computational pipeline started with a narrow filtering of hexapeptides based on their chemical similarity with known GPR120 agonists. The best hits were tested through docking studies, molecular dynamics and umbrella sampling simulations, which pointed to G[I,L]FGGG as a promising GPR120 agonist sequence. The presence of both peptides in food-related proteins was thoroughly assessed, revealing they may occur in mushrooms, food-grade bacteria and rice. Simulations on the counterparts with D-amino acids were also performed. Umbrella sampling simulations described that GdIFGGG may have a better interaction compared to its all-L counterpart (−13 kCal/mol ΔG and −6 kCal/mol ΔG, respectively). Overall, we obtained a predictive model to better understand the underpinning mechanism of GPR120-hexapeptides interaction, hierarchizing novel potential agonist peptides for further analysis and describing promising food sources worth of further dedicated investigations.

## Highlights

• GPR120 is involved in fatty taste perception and in many bioactivities.
• More than 500 000 peptides have been screened to mine novel GPR120 binders.
• G [I,L]FGGG were computed as promising agonist sequences (ΔG of GIFGGG -6 kCal/mol).
• D-amino acids-may boost the peptide-GPR120 interaction (ΔG of GdIFGGG -13 kCal/mol).
• Food-grade organisms were described as possible sources worthy of further analysis.

## Graphical abstract

**Figure:**
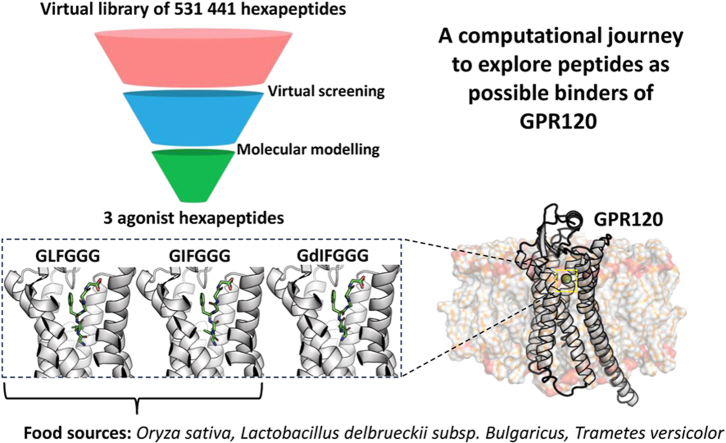

## Abbreviations used

DPPC: dipalmitoylphosphatidylcholine
FFAR4: Free Fatty Acid Receptor 4
GIP: gastric inhibitory peptide
GLP1: glucagon-like-peptide-1
GPCRs: G protein-coupled receptors
FFAs: free fatty acids
FFARs: free fatty acids receptors
CMD: conventional molecular dynamics
RMSD: Root Mean Squared Deviation
US: umbrella sampling

## 1 Introduction

Heterotrimeric G protein-coupled receptors (GPCRs), also known as 7-transmembrane domain receptors, are the largest family of cell-surface and signal transducing proteins (Civciristov et al., 2018) (up to 800 members identified so far) (Guo et al., 2022). They are integral transmembrane proteins and mediate most cellular responses to hormones and neurotransmitters. GPCRs also play a crucial role in taste and smell perceptions as they take a part in transducing the signal for aroma compounds as well as for sweet, bitter, umami, and kokumi taste (Ahmad and Dalziel, 2020). They have been also recently described as involved in the perception of the still debated fatty taste upon binding of free fatty acids (FFAs) (Galindo et al., 2012).

Several FFAs receptors (FFARs) were identified among GPCRs. Specifically, long-chain (>12 carbon atoms) saturated and unsaturated fatty acids bind and activate GPR40 and GPR120, while short-chain fatty acids (<6 carbon atoms; acetic acid, propionic acid and butyric acid, mainly) activate GPR41 and GPR43 (Kimura et al., 2020). A lot of studies focused on GPR120, also named Free Fatty Acid Receptor 4 (FFAR4; UniProt ID Q5NUL3), being an interesting drug target for the management and treatment of a series of disorders including Type 2 diabetes mellitus, obesity and those related to the sense of satiety (Burns and Moniri, 2010). GPR120 is relevantly expressed in enteroendocrine cells where it is involved in the secretion of glucagon-like-peptide-1 (GLP1) and cholecystokinin, known to play important roles in satiety and general food intake (Hirasawa et al., 2005). Further evidence pointing to its involvement in the antidiabetic action came in 2010 with the discovery of ω-3 fatty acids as suitable ligands (Hirasawa et al., 2005), which may associate with anti-inflammatory effects and consequent insulin sensitizing action (Oh et al., 2010). From a molecular standpoint, there are two human GPR120 isoforms, a long one (377 aa) and a short one (361 aa), differing for the insertion of 16 amino acids between positions 231 and 247 of the third intracellular loop. However, there is no evidence pointing to functional differences between the two isoforms which are meant to be functionally comparable (Pal et al., 2021).

As said before, FFAs are among the natural agonists of GPR120. However, the growing interest in this pharmacological target is driving the identification of new agonists for further developments either from a medicinal chemistry or food science standpoints – the former to derive pharmacologically active compounds, the latter to study the chemistry of fatty taste perception and/or to formulate nutraceuticals and functional foods. Several GPR120 agonists were identified over the years, such as TUG-891 and GW9508 (Hudson et al., 2013). Specifically, they enhance gastric inhibitory peptide (GIP) and GLP-1 secretion, suggesting that GPR120 may represent a valid antidiabetic drug target (Carullo et al., 2021; McKillop et al., 2023), as well as a target heading to anti-inflammatory processes (Anbazhagan et al., 2016).

Nowadays, besides non-natural pharmaceuticals, peptides are rising in popularity as therapeutics to target proteins and pathways involved in the onset and progression of certain diseases (Davenport et al., 2020) as well as to design food supplements and nutraceuticals (de Castro and Sato, 2015). Indeed, bioactive peptides derived from food proteins upon processing (e.g. via hydrolysis and fermentation) were shown to have potential applications as ingredients in functional foods owing to their potential health-promoting characteristics (Udenigwe and Aluko, 2012). Similarly, food rich in bioactive peptides may bring benefit when properly consumed (Udenigwe and Aluko, 2012). This makes the identification of bioactive sequences and their organism sources relevant from a food science standpoint. Germane to GPCRs, several agonist peptides have been already described (Davenport et al., 2020; Krumm and Grisshammer, 2015). Nonetheless, the discovery of new agonist peptides is relevant either to expand the chemical space of GPCRs ligands (fundamental for drug development) or to find valuable food-grade protein sources to develop nutraceuticals and functional foods.

In this framework, the present study aimed at investigating the mechanics of GPR120-ligand interaction to identify new potential GPR120 peptide ligands. To do so, a virtual library of 531 441 hexapeptides has been set up and screened to mine sequences showing chemical analogies to known GPR120 ligands (namely, oleic acid and TUG-891), based on the assumption that similar compounds may activate the same target (McKinney et al., 2000). Afterward, a selection of the best hits underwent a 3D molecular modelling study integrating docking, conventional molecular dynamics (CMD) and umbrella sampling (US) simulations to thoroughly estimate the receptor activation, in agreement with previous studies (Lammi et al., 2021; Vidal-Limon et al., 2022).

Overall, this study developed a predictive model to better understand the underpinning mechanism of GPR120-hexapeptides interaction, hierarchizing novel potential agonist peptides and their food-related sources for further analysis. The effect of D-amino acids in the activity of those sequences proposed to be active has been also evaluated.

## 2 Materials and methods

### 2.1 GPR120 model construction

The 3D model of GPR120 was derived from the crystallographic structure available in Protein Data Bank (https://www.rcsb.org) (Berman et al., 2000) with the PDB code 8ID6 (Chain A) (Mao et al., 2023). The structure was chosen based on its good resolution (2.80 Å), although it was uncomplete in some parts (residues 1–22, 33–34, 70–74 and 145–150). The structural continuity was obtained by homology modelling using the SwissModel tool (https://swissmodel.expasy.org/) (Waterhouse et al., 2018). SwissModel is a structural bioinformatic web server for generating 3D models of proteins using a comparative approach. The 3D structure 8ID6 was used as template while the FASTA sequence of human GPR120 (Uniprot code: Q5NUL3) was used as input. After that, the predicted structure was superimposed to the AlphaFold (https://alphafold.ebi.ac.uk/) (Jumper et al., 2021) structure and residues 15–30 were substituted with those of the AlphaFold model as their reconstructions seemed more plausible, in agreement with previous studies (Dellafiora et al., 2020).

### 2.2 Retrieval of ligands 3D information

The structures of TUG-891 and FFAs were retrieved from PubChem (https://pubchem.ncbi.nlm.nih.gov/) (Kim et al., 2023) in the Structured Data File (.sdf): TUG-891 (CID: 57522038), oleic acid (CID: 445639), isobutyric acid (CID: 6590) and propionic acid (CID: 1032). Then, they were converted to the Tripos Mol2 format (.mol2) using Open Babel (O'Boyle et al., 2011) to enable further analysis.

### 2.3 Construction of peptides libraries

The 3D hexapeptides structures analyzed in this study were built using an *ad hoc* python script interfaced with PyMol (opensource v. 2.3.0; https://pymol.org), in agreement with a previous study (Dellafiora et al., 2022). All the possible combinations of 9 non-polar amino acids (i.e. glycine, alanine, leucine, isoleucine, valine, methionine, proline, phenylalanine and tryptophan) were generated, for a total of 531 441 combinations. The protonation state was set at pH 7 with C- and N- terminal as deprotonated and protonated, respectively. The entire set of generated peptides was collected in the Tripos Mol2 format. The list of peptides including D-amino acid was generated by manually editing the respective amino acid position using the *invert* PyMol's function.

### 2.4 Ligand-based virtual screening of the library

The hexapeptides library underwent a ligand-based virtual screening to mine peptides according to their similarity to oleic acid and TUG-891, taken as reference for GPR120 natural and synthetic ligands, respectively. The ligand-based virtual screening was performed using the LiSiCA (Ligand Similarity using Clique Algorithm) algorithm (Legnik et al., 2015) as previously succeeded to rank entries to efficiently estimate their bioactivity (Del Favero et al., 2022; Dellafiora et al., 2022). This algorithm provides a fast ligand-based virtual screening platform to quantify chemical similarities between a reference template (oleic acid and TUG-891 in this case) and a database of target compounds. LiSiCA expresses similarities using the Tanimoto coefficient's metric, a gold standard to quantify chemical analogies (from 0 to 1, with 1 meaning identical molecules). LiSiCA's default parameters were used. This fast virtual screening aimed to identify a small selection of hexapeptides within the whole library to carry forth to the molecular modelling steps.

### 2.5 Docking simulations

Molecular docking simulations aimed to provide a plausible binding architecture for TUG-891, FFAs and hexapeptides obtained from the virtual screening and were performed using the GOLD software (v. 2021). The binding site was defined within a 10 Å radius sphere from the centroid of the pocket. The water #501 (from PDB structure 8ID4) was included and set forming hydrogen bonds (default settings) as it was described involved in polar interactions with ligands (Mao et al., 2023). Additionally, a spatial constraint has been used to better reproduce the binding architecture as per crystallographic data setting the similarity shape overlap option (weight 100) with respect to the crystallographic pose of oleic acid in 8ID6. According to previous study (Pedroni et al., 2023), a semi-flexible docking approach was applied keeping ligands fully flexible and the protein semi-flexible allowing polar hydrogens free to rotate. 10 poses for each ligand were generated and the internal score function PLPScore was used to estimate the fitting of each ligand within GPR120 binding site (the higher the score, the more likely the predicted binding architecture; https://www.ccdc.cam.ac.uk) as it already proved to reliably reproduce architectures of binding (Del Favero et al., 2022).

### 2.6 Conventional molecular dynamics simulations

CMD simulations were performed using GROMACS (version 2021.4) (Abraham et al., 2015) to study ligands-GPR120 stability over time. Before running CMD, the protein structure was embedded into a dipalmitoylphosphatidylcholine (DPPC) membrane using the CHARMM-GUI (Jo et al., 2008; Lee et al., 2016) membrane builder tool (https://www.charmm-gui.org). More in detail, CHARMM-GUI is a web-based user interface which allows to build realistic biological membrane systems. The whole system was parametrized with CHARMM36 all-atom force field (Lee et al., 2016). TUG-891, oleic acid, isobutyric acid and propionic acid parametrization was performed on the SwissParam webserver (https://www.swissparam.ch) (Zoete et al., 2011). Each complex was solvated with SPCE waters in a cubic periodic boundary condition and the system was neutralized by adding counter ions (Na^+^ or Cl^−^). After that, each system was energetically minimized to avoid steric clashes and to correct improper geometries using the steepest descent algorithm with a maximum of 5000 steps. Next, all the systems underwent isothermal (300 K, coupling time 2 ps) and isobaric (1 bar, coupling time 2 ps) 100 ps simulations before undergoing a 40 ns CMD simulation each.

### 2.7 Umbrella sampling simulation

The protein-peptide complex with the highest PLPScore was chosen for further investigations through US. Simulations were performed using GROMACS (version 2021.4) to estimate protein-peptide binding free energy and the whole system was parametrized with CHARMM36 all-atom force field (Huang and MacKerell, 2013). The input structure was placed in a rectangular box with dimensions sufficient to provide space for the pulling simulations to take place along the Y-axis. The box was then solvated with SPCE water and neutralized by adding counter ions (Na^+^ and Cl^−^). Subsequently the system was energetically minimized with a maximum of 50 000 steps and underwent isothermal (310 K, coupling time 0.1 ps) and isobaric (1 bar, coupling time 2 ps). The peptide was then pulled from the protein binding site at 0.01 nm/ps pull rate over the course of 500 ps on the Y-axis using a spring force constant of 1500 kJ mol^−1^ nm^−2^. The frames obtained from the pulling were used as initial coordinates for binding free energy calculations through the US simulations. An asymmetric distribution of sampling windows was used such that spacing along the reaction coordinate was 0.2 nm. Each US window underwent an NPT equilibration before running a 10 ns simulation with a spring force constant of 1500 kJ mol^−1^ nm^−2^.

### 2.8 Identification of potential proteins source of food origin and possible release

An iterative sequence alignment procedure was set up based on an *ad hoc* bash script. This was meant to search each of the most promising hexapeptides (namely, GIFGGG and GLFGGG) within the whole SwissProt database (570 420 protein sequences; last database access December 6, 2023). Briefly, SwissProt is the manually curated part of UniProt (https://www.uniprot.org) (Bateman et al., 2023) collecting all the protein sequences referenced as “reviewed” (i.e. derived from scientific literature or, if computationally derived, curated and evaluated by an expert analysis).

## 3 Results and discussion

### 3.1 Model validation

Previous works demonstrated that the procedural workflow used here may reliably estimate whether a given molecule may bind and activate a designated receptor (Dellafiora et al., 2022; Pedroni et al., 2023; Wu et al., 2022). Specifically, docking simulations, which aimed at providing plausible binding architectures scored based on their fitting into the pocket, were integrated to CMD and US simulations to study the complex stability and energy over time. Of note, monitoring the geometrical stability of the receptor-ligand complex over time may efficiently estimate the activity of ligands. Specifically, a ligand is meant to be an agonist when its interaction with the receptor is stable over time, preserving at the same time the overall structure of the given activated receptor. Conversely, unstable complexes, e.g. with ligands that can not persist at the designated binding site and/or when the overall protein structure loses the native (active) structure, point to the incapability of ligands to appreciably activate the receptor. The capability of *in silico* methods to achieve such analysis for GPCRs and peptides has been already demonstrated (Dellafiora et al., 2022; Pedroni et al., 2023). However, a fit-for-purpose assessment of procedural performances has been done to define the reference scenario for active ligands and for molecules unable to appreciably bind and activate the receptor. In this respect, oleic acid and TUG-891 were taken as positive controls being well-known GPR120 agonists, while propionic and isobutyric acid were taken as negative controls, being unable to bind and activate GPR120 (Galindo et al., 2012).

Once defined the set of reference compounds, they were docked at the receptor binding site and the calculated poses of positive controls (oleic acid and TUG-891) were compared to the respective crystallographic poses (as per PDB structure with code 8ID6 and 8ID8, respectively). As shown in Fig. 2, all the molecules considered engaged the receptor with a similar binding pose, arranging the carboxylic acid moiety as per the crystallographic pose of oleic acid (PDB code 8ID6). Particularly, for positive controls, calculated and crystallographic poses were comparable proving the model effectiveness to reliably reproduce the binding pose of ligands. Of note, as shown in Table 1, the negative controls recorded scores lower than the known GPR120 ligands TUG-891 and oleic acid. Although this might have been due to the diverse number of atoms (short-chain FFAs have inherently a lower number of atoms compared to the mid-long counterpart), the score assignment could suggest which molecules were able to best fit the receptor pocket (the higher the score, the better the fitting, in agreement with the manufacturer declarations; https://www.ccdc.cam.ac.uk). Then, the four complexes underwent CMD simulations to monitor their stability over time. As shown in Fig. 2 and S1(see Supplementary Materials), the negative controls (i.e. isobutyric acid and propionic acid) could not stably interact at the ligand binding site showing a full detachment from the designated interaction site within 25 ns. Conversely, the positive controls (i.e. oleic acid and TUG-891), remained stable at the ligand binding site, as shown by the root mean squared deviation (RMSD) analysis of the carboxylate moiety and the respective trajectories. They also kept the overall receptor structure stable. Of note, RMSD fluctuation was monitored only for the carboxylate moiety with respect to the whole protein to enable a comparative analysis among the ligands considered (including peptides), as they differ in shape and number of atoms (see Fig. 2).

**Table 1:** Virtual screening and docking scores.

Ligands		LiSiCA Score^a^	PLPScore^b^
FFAs and synthetic agonist	Propionic acid	n.p.	95.4
	Isobutyric acid	n.p.	98.0
	Oleic acid	n.p.	143.8
	TUG-891	n.p.	162.0
Total L peptides	**GLFGGG**	**0.281**	**145.0**
	**GIFGGG**	**0.281**	**151.5**
	GGPGWG	0.280	135.4
	GIGGGA	0.282	140.2
	GIGWGA	0.282	128.9
	GIPGWG	0.284	119.3
	GLGGGA	0.282	131.9
	GLGWGA	0.282	132.5
	GLPGWG	0.284	115.7
Peptides with D-amino acids	GdIdFGGG	n.p.	142.7
	**GdIFGGG**	n.p.	**156.9**
	GdLdFGGG	n.p.	121.4
	GdLFGGG	n.p.	147.2
	GLdFGGG	n.p.	146.4
	GIdFGGG	n.p.	146.2

*Note*: ^a^ average LiSiCA score using alternatively TUG-891 and oleic acid as reference compound; ^b^ The higher the score, the better the pocket fitting, as per manufacturer declaration (https://www.ccdc.cam.ac.uk); peptides considered for molecular dynamics are in bold; n.p. Stands for not performed.

Taken together, these outcomes showed that the integrated use of docking analysis and CMD can reliably distinguish GPR120 ligands from non-ligands, with geometrical parameters like RMSD and trajectories analysis as probative features to enable ligands discriminations and function assignment.

### 3.2 Analysis of peptides library

#### 3.2.1 Virtual screening

**Fig. 1:**
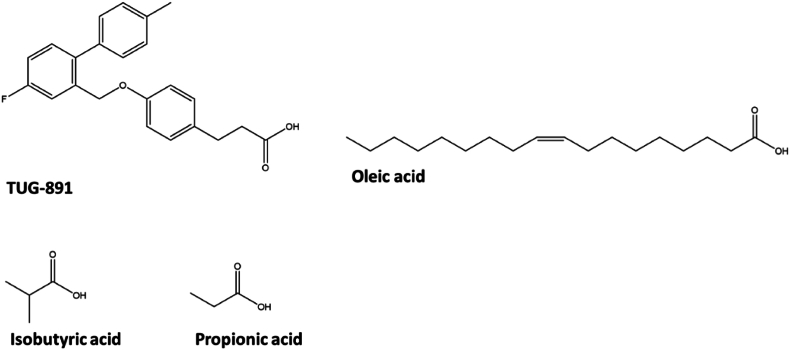
Chemical structures of positive (TUG-891 CID 57522038, and oleic acid CID 445639) and negative (isobutyric acid CID 6590, and propionic acid CID 1032) controls.

The 3D hexapeptides library analyzed in this study was built using an *ad hoc* python script interfaced with PyMol generating all the possible combination of the 9 non-polar amino acids (see section 2.3). This was done to obtain peptides with a partial chemical similarity in terms of steric properties with the native GPR120 binders (i.e. long chain fatty acids). The hexapeptides library screening was achieved using the LiSiCA algorithm (Legnik et al., 2015) setting default parameters (see section 2.4). Two different screening procedures were run setting alternatively oleic acid (i.e. the natural substrate retrieved from the crystallographic structure with PDB ID 8ID6) and TUG-891 (i.e. the synthetic agonist retrieved from the crystallographic structure with PDB ID 8ID8) as reference compounds (Fig. 1). As expected, the significant chemical difference between the hexapeptides and the reference compounds led to relatively low scores. This was not considered a limiting factor for the sake of this study since this step was crucial to obtain a feasible number of hexapeptides to enable the subsequent molecular modelling analysis.

**Fig. 2:**
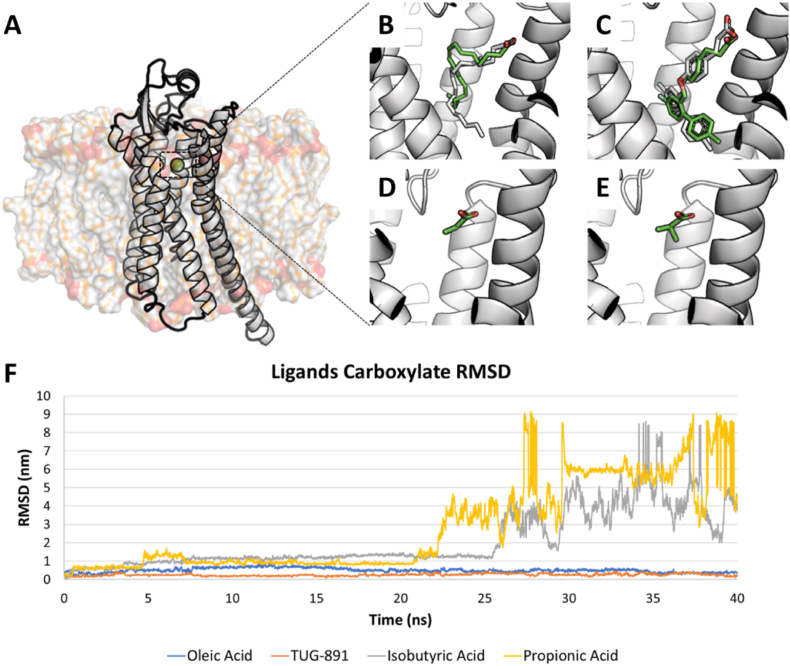
Validation of GPR120 model via molecular docking and dynamic simulations. **A**. GPR120 3D model represented as white cartoon within membrane represented as semi-transparent surface. The green sphere represents the centroid set for the docking simulation. **B**. Oleic acid best-scored docking pose (green sticks) superimposed to oleic acid retrieved from PDB ID 8ID6 (white sticks). **C**. TUG-891 best-scored docking pose (green sticks) superimposed to TUG-891 retrieved from the PDB ID 8ID8 (white sticks). **D**. Propionic acid best-scored docking pose (green sticks) **E**. Isobutyric acid best-scored docking pose (green sticks). **F**. Ligands carboxylate RMSD representing the distance of the carboxylate moiety from the protein. Of note, both isobutyric and propionic acid detached from the GPR120 binding pocket within 25 ns.

The average score from the two screening procedures was computed for each hexapeptide obtaining their overall ranking via an *ad hoc* python script. Arbitrarily, only hexapeptides scoring equal or higher than 0.28 units were considered to focus the analysis on those most similar to the reference compounds. This resulted in a list of 9 hexapeptides: GLFGGG, GIFGGG, GGPGWG, GIGGGA, GIGWGA, GIPGWG, GLGGGA, GLGWGA and GLPGWG (Table 1).

#### 3.2.2 Analysis of best hits

**Fig. 3:**
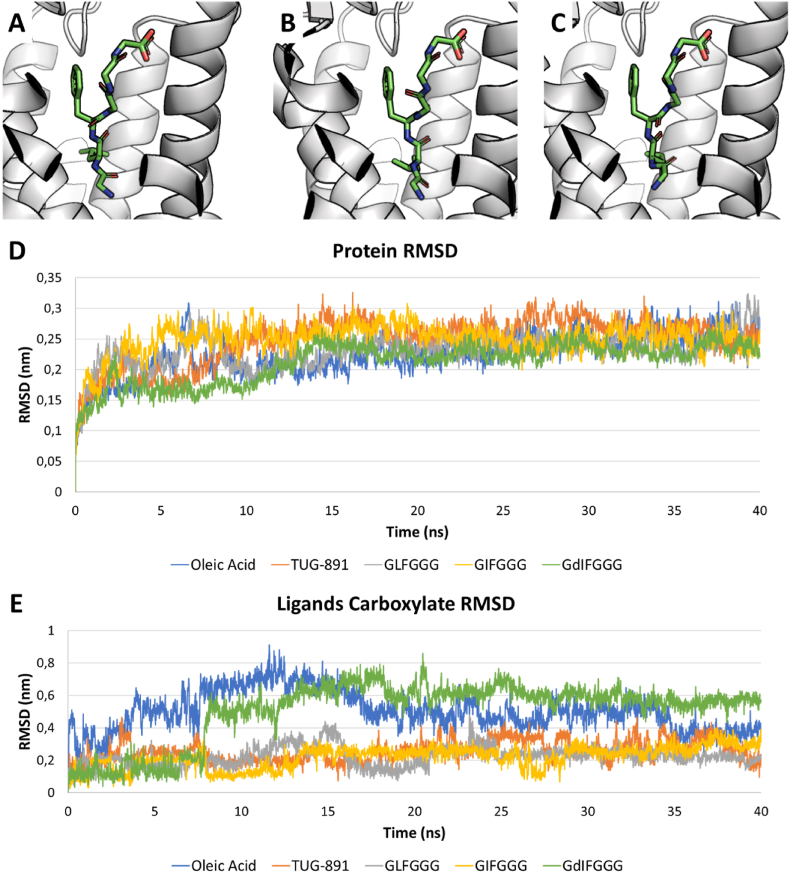
Molecular docking and CMD simulations results for GLFGGG, GIFGGG and GdIFGGG. **A**. Best-scored docking pose for GLFGGG represented as green sticks. **B**. Best-scored docking pose for GIFGGG represented as green sticks. **C**. Best-scored docking pose for GdIFGGG represented as green sticks. **D**. Protein RMSD graph comparing the positive controls (i.e. oleic acid and TUG-891) with GLFGGG, GIFGGG and GdIFGGG. As appreciable from the graph, the protein RMSD trends are comparable. **E**. Ligands carboxylate RMSD representing the distance of the carboxylate moiety from the protein. All the tested ligands showed a stable trend within the GPR120's binding pocket although GdIFGG (green) showed an initial movement which stabilized around 8 ns from the beginning of the CMD onward. (For interpretation of the references to color in this figure legend, the reader is referred to the Web version of this article.)

**Fig. 4:**
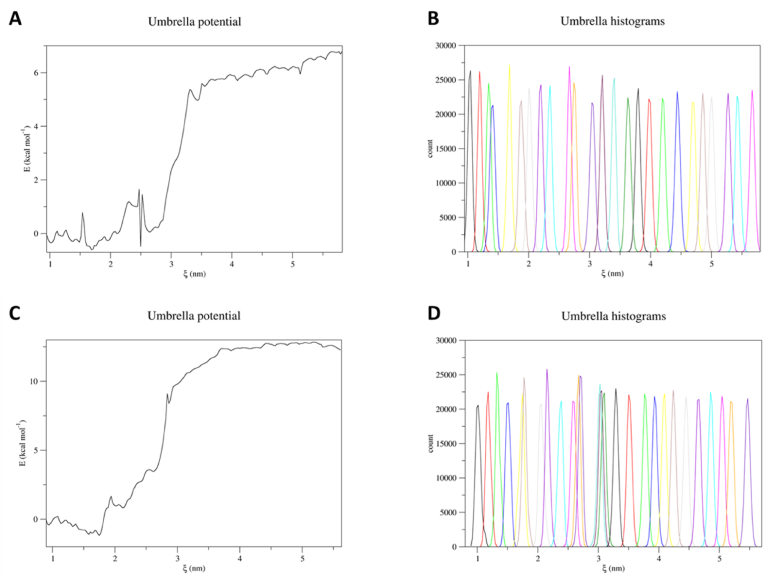
US results of the GIFGGG and GdIFGGG peptides. **A**. GIFGGG umbrella potential graph. **B**. GIFGGG histograms distribution showing total coverage of the pulling procedure. **C**. GdIFGGG umbrella potential graph. **D**. GdIFGGG histograms distribution showing total coverage of the pulling procedure.

Based on the above, and on the assumption that molecules sharing analogies in terms of molecular structure may target the same protein (McKinney et al., 2000), the following peptides underwent docking analysis to estimate their actual capability to interact with GPR120: GLFGGG, GIFGGG, GGPGWG, GIGGGA, GIGWGA, GIPGWG, GLGGGA, GLGWGA and GLPGWG. As shown in Table 1, GLFGGG and GIFGGG recorded the highest docking scores (PLPScore of 145.0 and 151.5 units, respectively). This suggested that they may be those most able to fit the receptor pocket among those considered in docking studies (the higher the score, the better the interaction). Afterward, they underwent CMD to check the peptide-receptor complex stability over time to compare the outcome with the reference scenario obtained for positive and negative controls (see section 3.1), predicting their possible activity thereby. As shown in Fig. 3 and S2 (see Supplementary Materials), the RMSD and trajectory analysis described that they both kept a steady interaction at the designated binding site of GPR120 keeping the overall protein structure stable over time. This pointed to the possible capability of both peptides to effectively interact and activate GPR120, as proved in previous studies for many other peptide-activated receptors. Specifically, peptides have been already described as able to activate GPCRs involved in taste perception, as found for e.g. umami, bitter and kokumi (Dellafiora et al., 2022; Wang et al., 2022; Yu et al., 2022). To further verify this hypothesis, an US simulation was performed to estimate the free energy of binding for a thorough assessment of complex stability. Since both the hexapeptides showed comparable results in terms of CMD simulations, the US simulation was performed only on the GIFGGG peptide as it obtained the highest docking score (i.e. 151.5 units). Once GIFGGG was pulled out for 5 nm, 24 windows with a 0.2 nm interval were chosen to perform the US simulation (see section 2.6). This enabled the collection of histograms and the umbrella potential graph reported in Fig. 4. The peaks distribution in the histogram reflected the good sampling obtained via the parameters set (section 2.6). Indeed, the evenly spaced windows were sufficient to cover all the regions GIFGGG passed by, exhibiting a satisfactory overlap. The analysis of the umbrella potential graph showed a ΔG corresponding to roughly −6 kcal/mol, pointing to its energetically favorable interaction with GPR120, in line with other studies on protein-peptide complexes (Cool and Lindert, 2022). Of note, the capability of hexapeptides to activate a GPCR described here is in line with previous finding describing identical length peptides able to activate a variety of GPCRs, including those associated with taste perception (Calderón et al., 2023; Thibeault et al., 2020; Wang et al., 2022). This evidence further confirmed supported the hypothesized appreciable binding of GIFGGG with GPR120.

This outcome is crucial since the identification of peptides able to interact and activate GPR120 would have many implications in terms of receptors pharmacology and related medicinal chemistry, as well as from a food science standpoint. As an example, the possible existence of GPR120 peptide agonists may significantly expand the chemical space of GPR120 ligands. In this respect, agonist peptides have not been described yet for GPR120. This is reasonably due to its relatively recent de-orphanization, though the existence of agonist peptides could be expected based on the growing evidence of peptides with agonists activity towards other GPCRs related to GPR120 (Davenport et al., 2020). The characterization of agonist peptides is also important toward a more informed understanding of fatty acid receptors pharmacology (including GPR120) to unlock their ultimate therapeutic potential and pharmacological intervention routes (Milligan et al., 2017). Regarding this case study, GPR120 has a role in several diseases, including cancer, inflammatory conditions, central nervous system disorders and Type 2 diabetes mellitus (Carullo et al., 2021). Moreover, GPR120 is abundantly expressed in entero-endocrine cells, adipocytes, taste buds of circumvallate, fungiform, and foliate papillae, and through the gastrointestinal tract (Carullo et al., 2021; Lu et al., 2018; Miyamoto et al., 2016). This could suggest a first-line role in the complex network of molecular events underpinning the action of food bioactive constituents. Therefore, from a pharmacological standpoint, the two sequences identified here could suggest a promising scaffold to investigate further and derive compounds with enhanced pharmacological properties (as sub-optimal pharmacodynamics and pharmacokinetics are typically expected for naturally occurring peptides (Davenport et al., 2020)). From a food science perspective, the identification of peptides able to activate GPR120 deserves attention considering the multifaceted role it has been ascribed to, and consequent plausible implications in the food-health binomial. Specifically, further investigations of GLFGGG and GIFGGG shall reveal to what extent they effectively activate GPR120 in a real-world scenario. This may promote a thorough identification of GPR120 agonist peptides of food origin from a foodomics perspective – referred to as the comprehensive, high-throughput fingerprinting of food bioactives with a role to improve human nutrition and wellbeing (Capozzi and Bordoni, 2013). In this respect, the identification of GPR120 agonist peptides should be systematically pursued considering that they typically have an individual limited pharmacological activity, but the complex mixture of peptides of food origin may reach pharmacologically relevant concentrations as a chemical complex rather than single substances. In addition, the possible involvement of GPR120 in the perception of the so called “fatty taste” (Iwasaki et al., 2021) may lead to consider selected peptides as useful scaffold to derive fatty taste elicitors. On the other side, a deeper understanding of peptides-GPR120 interaction and subsequent activation may shed light on the chemistry of taste toward a more informed profiling of taste active components of food. The above makes the systematic and comprehensive study of GPR120 agonist peptides of food origin critical. In this respect, the sequences identified here described hexapeptides rich in glycine as the prototype scaffold for agonist peptides of GPR120 and provided a compelling line of evidence pointing to the need of moving pioneering steps to instruct knowledge-based research of new peptide-based GPR120 ligands.

### 3.3 Identification of possible sources of food origin for GLFGGG and GIFGGG

As discussed above, the identification of possible food sources of GLFGGG and GIFGGG is crucial to move the outcome presented to a real-world scenario. This may be important either to characterize better the chemical complex of certain foods possibly responsible for the biological outcomes associated with their consumption, or to identify valuable sources of bioactives to derive e.g. functional foods or nutraceuticals (Lammi et al., 2021).

Germane to the identification of possible sources of GLFGGG and GIFGGG, both sequences have been searched within the whole SwissProt database (570 420 protein sequences; last database access December 6, 2023) (Bateman et al., 2023) collecting information about “reviewed” sequences (i.e. derived from scientific literature or, if computationally derived, curated and evaluated by an expert analysis). The focus on the SwissProt database, rather than extending the analysis over the whole UniProt database, aimed at pursuing the identification of reasonably existing proteins, excluding those hypotheticals from the analysis (Bateman et al., 2023).

As shown in Table 2, 36 diverse UniProt entries from several organisms, including procaryotes and eukaryotes, were found to contain GLFGGG (27 entries) or GIFGGG (9 entries). The relative shortage of proteins found is in line with the particularity of the two sequences under analysis. Indeed, polyglycine segments are not functionally neutral and they are typically associated with poorly organized protein regions and/or signals which may impact protein function, folding, transport and turnover (Bykov and Asher, 2010; Endow et al., 2016). For this reason, its frequency is expected to be low. Among those identified, three were of possible interest from a food standpoint: endoglucanase 4 from *Oryza sativa* (rice), ferredoxin--NADP reductase from *Lactobacillus delbrueckii* subsp. *Bulgaricus* (lactic acid bacteria common in foodstuff)*,* and ligninase C from *Trametes versicolor* (a mushroom not edible *per se* but considered for food supplements production). These are possible valuable sources from a food perspective as rice is massively consumed worldwide with many uses also as food supplement, *L. delbrueckii* is used in a large variety of fermented milk products, and *T. versicolor*, which is not considered edible for humans though it is used to manufacture food supplements (Barros et al., 2016). However, it was not possible to retrieve from the literature clear information about the level of expression of rice endoglucanase 4 and bacterial ferredoxin—NADP reductase within the respective organism of origin. Therefore, the relevance of GIFGGG/GLFGGG-containing proteins and related organisms from a food production standpoint could not be ascertained. Conversely, ligninase C is a key enzyme of *T. versicolor* expected to be highly expressed as it is crucial for lignin degradation, a fundamental step in the physiology and nourishment of this fungus (Jonsson and Nyman, 1992). Of note, *T. versicolor* has been already described as a promising organism rich in health-promoting components, and it has been also considered as an ingredient for food supplements (Barros et al., 2016; Janjusevic et al., 2017, 2018). Therefore, besides considering the pure sequence obtained via chemical synthesis, the consumption of food supplements enriched with or made of *T. versicolor* might be a possible valuable source of GIFGGG worth of further dedicated analysis.

**Table 2:** Possible protein sources of GLFGGG and GIFGGG.

UniProt ID	Organism	Protein
Q96L46^a^	*Homo sapiens*	Calpain small subunit 2
A5U7B6^a^	*Mycobacterium tuberculosis*	Cell division protein FtsX
P9WG18^a^		
P9WG19^a^		
Q7TX91^a^	*M. bovis*	
O32882^a^	*M. leprae*	
P27747^a^	*Cupriavidus necator*	Dihydrolipoyllysine-residue acetyltransferase component of acetoin cleaving system
A6L2J8^b^	*Phocaeicola vulgatus*	Dipeptidyl-peptidase 7
Q5LB17^b^	*Bacteroides fragilis*	Dipeptidyl-peptidase 7
B4UCY7^a^	*Anaeromyxobacter* sp. *(strain K)*	DNA mismatch repair protein MutS
B8JA66^a^	*A. dehalogenans*	
Q2IIJ3^a^		
**Q6Z715**^**b**^	*Oryza sativa* subsp. *Japonica*	Endoglucanase 4
**Q049B3**^**b**^	*Lactobacillus delbrueckii* subsp. *Bulgaricus*	Ferredoxin--NADP reductase
**Q1G967**^**b**^		
Q6Q8A5^a^	*Nicotiana tabacum*	Hexokinase-2
P13645^b^	*H. sapiens*	Keratin, type I cytoskeletal 10
**P20013**^**b**^	*Trametes versicolor*	Ligninase C
P91928^a^	*Drosophila melanogaster*	MICOS complex subunit Mic 60
Q9VCH5^a^	*D. melanogaster*	Nuclear pore complex protein Nup98-Nup96
O82230^a^	*Arabidopsis thaliana*	Nucleoid-associated protein At2g24020
Q9M098^a^	*A. thaliana*	Nucleoid-associated protein At4g30620
G0SAK3^a^	*Chaetomium thermophilum*	Nucleoporin NUP145
Q9UTK4^a^	*Schizosaccharomyces pombe*	Nucleoporin nup189
B2HE92^a^	*M. marinum*	PE cleavage protein A
L0T4W6^b^	*M. tuberculosis*	PE-PGRS family protein PE_PGRS4
Q2NT27^b^	*Sodalis glossinidius*	Phenylalanine--tRNA ligase beta subunit
E1XTG6^a^	*Salmonella phage ViI*	Portal protein
P0CQ46^a^	*Cryptococcus neoformans*	Protein SEY1
P0CQ47^a^		
Q9WU70^a^	*Rattus norvegicus*	Syntaxin-binding protein
Q5T5C0^a^	*H. sapiens*	
Q8K400^a^	*Mus musculus*	
Q54LC9^a^	*Dictyostelium discoideum*	Uncharacterized Golgi apparatus membrane protein-like protein
Q9HDZ8^a^	*S. pombe*	Uncharacterized protein C589.06c
Q9XJR4^a^	*Pseudoalteromonas phage PM2*	Uncharacterized protein Gp-j

*Note.*^a^ Presence of GLFGGG; ^b^ Presence of GIFGGG. In bold those considered relevant from a food perspective.

### 3.4 D-amino acid containing derivatives of GIFGGG

It has been previously demonstrated that the inclusion of D-amino acid in peptides may significantly enhance their resistance to proteases activity (Yan et al., 2020). Therefore, calculations of D-amino acid containing derivatives of GIFGGG and GLFGGG were added for the sake of estimating the possible activity of derivatives likely more resistant to gastrointestinal digestion. Particularly, all the possible combinations including one or two D-amino acids in the two sequences were analyzed (Table 1). As shown in Table 1, one out of the six combinations possible (namely, GdIFGGG) recorded a docking score higher than the parental peptide suggesting a better fitting into the pocket and possibly a higher activity toward GPR120. Therefore, the GdIFGGG-GPR120 complex underwent CMD and US to estimate the geometrical stability over time and the free energy of binding. As shown in Fig. 3 and S2 (see Supplementary Materials), the RMSD of the carboxylate moiety with respect to the protein was higher than the corresponding L-peptide. However, this was due to an initial adjustment of the starting binding pose which eventually stabilized on a trend comparable to its L counterpart. Moreover, as shown in the protein RMSD plot (Fig. 3), it kept the overall protein structure as stable as the complexes with positive controls (i.e. oleic acid and TUG-891). Regarding the US simulation, once GdIFGGG was pulled out for 5 nm, 26 windows with an approximate 0.2 nm interval were chosen to perform the US simulation (see section 2.6). The collection of histograms and the umbrella potential graph are shown in Fig. 4. The peaks distribution in the histogram reflected the total coverage for the pulling simulation obtained via the parameters set (section 2.6). Indeed, the evenly spaced windows were sufficient to cover all the regions GdIFGGG passed by, exhibiting a sufficient overlap. The analysis of the umbrella potential graph showed a ΔG corresponding to roughly −13 kcal/mol, pointing to an interaction with GPR120 possibly more favored than that with its L counterpart. This was in line with other studies on protein-peptide complexes (Cool and Lindert, 2022). Moreover, previous evidence described that hexapeptides may bind GPCRs with a high affinity showing values of free energy of binding in the range of that obtained here for GdIFGGG (Calderón et al., 2023). The finding that D-amino acid containing sequences may have an enhanced activity is important in the light of the common production of D-amino acid during food processing. Indeed, they can be either present in raw materials (such as some fruits and vegetables) or produced upon processing like fermentation or the application of thermal treatments (Genchi, 2017; Marcone et al., 2020). Germane to our study, the design of proper treatments of *T. versicolor* in food supplements production may result in an enriched fraction of D-amino acid containing peptides, which are worth of future dedicated investigations.

## 4 Conclusion

The present work identified from a virtual library of more than 500 000 entries two hexapeptides, i.e. GLFGGG and GIFGGG, with a potential agonist activity toward GPR120 which are worth of further dedicated investigations. GPR120 is an intriguing target relevant to pharmaceutical and food science being involved in the fatty taste perception as well as in a wealth of biological outcomes reasonably germane to the mechanism of action of food bioactives. The identification of peptides as possible agonists of GPR120 is consistent with evidence for other GPCRs and may expand the chemical space of GPR120 ligands. In this respect, our results provided a convincing rationale supporting peptides as useful scaffold to derive new agonists in further dedicated studies. This may be important for food science as GPR120-agonist peptides and “natural” peptidomimetic compounds (such as D-amino acids containing derivatives) might be considered for food supplements/functional foods production or taste elicitors development. The outcomes presented also highlighted that certain food-related proteins may be a valuable source for the identified sequences. Finally, the analysis also covered D-amino acid containing sequences, which are reasonably more resistant to gastrointestinal digestion, and described GdIFGGG having a better interaction compared to its all-L counterpart (−13 kCal/mol ΔG and −6 kCal/mol ΔG, respectively). This may be important in the light of designing nutraceuticals or functional foods targeting GPR120.

## CRediT authorship contribution statement

**Lorenzo Pedroni:** Methodology, Formal analysis, Investigation, Writing – original draft, Writing – review & editing. **Florinda Perugino:** Methodology, Formal analysis, Investigation, Writing – original draft, Writing – review & editing. **Fabio Magnaghi:** Methodology, Formal analysis, Investigation, Writing – original draft, Writing – review & editing. **Chiara Dall’Asta:** Writing – review & editing, Conceptualization. **Gianni Galaverna:** Writing – review & editing, Conceptualization. **Luca Dellafiora:** Methodology, Formal analysis, Investigation, Writing – original draft, Writing – review & editing, Conceptualization, Supervision.

## Declaration of competing interest

The authors declare that they have no known competing financial interests or personal relationships that could have appeared to influence the work reported in this paper.

## Data Availability

Data will be made available on request.
