# Supplementary material for: Free fatty acid receptors beyond fatty acids: A computational journey to explore peptides as possible binders of GPR120
Source: Curr Res Food Sci. 2024 Mar 4;8:100710. doi: 10.1016/j.crfs.2024.100710 (PMC10940776; doi:10.1016/j.crfs.2024.100710)
Supplement: Multimedia component 1 [file mmc1.docx]

**Free fatty acid receptors beyond fatty acids: a computational journey to explore peptides as possible binders of GPR120**

Lorenzo Pedroni ^1,#^, Florinda Perugino ^1,2,#^, Fabio Magnaghi ^1^, Chiara Dall’Asta ^1^, Gianni Galaverna ^1^, Luca Dellafiora ^1,^*

^1^ Department of Food and Drug, University of Parma, Parma, Italy

^2^ Department of Biology, University of Naples Federico II, Naples, Italy

^#^ These authors contributed equally to the work

* Correspondence to: Luca Dellafiora, Department of Food and Drug, University of Parma, Parco Area delle Scienze 27/A, 43124 Parma, Italy. Phone: +39 0521 902073. Email: [luca.dellafiora@unipr.it](mailto:luca.dellafiora@unipr.it)

**
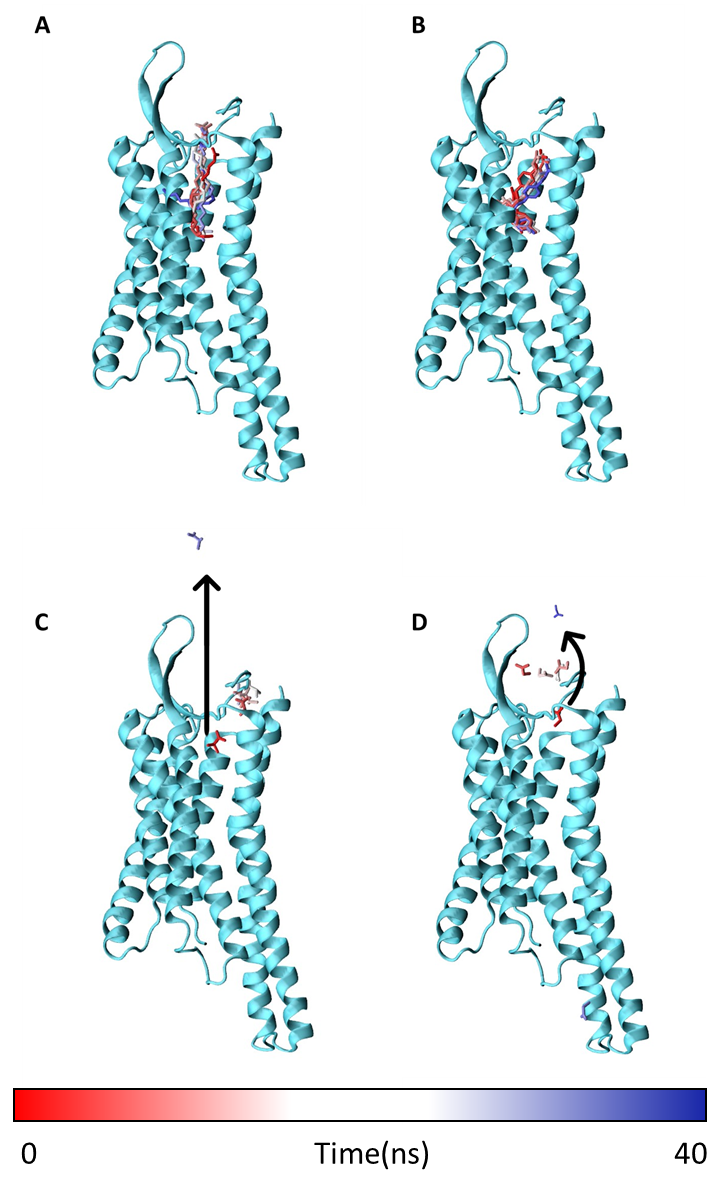
**

**Figure S1**. Trajectory of oleic acid (A), TUG-891 (B), isobutyric acid (C) and propionic acid (D) within GPR120 binding site. The protein is represented by cyan cartoon while each ligands by sticks. The red-to-blue transition reports the stepwise changes of coordinates for each ligand, represented as sticks, over the 40 ns CMD simulation. Of note, both isobutyric acid (C) and propionic acid (D) detached from the protein.


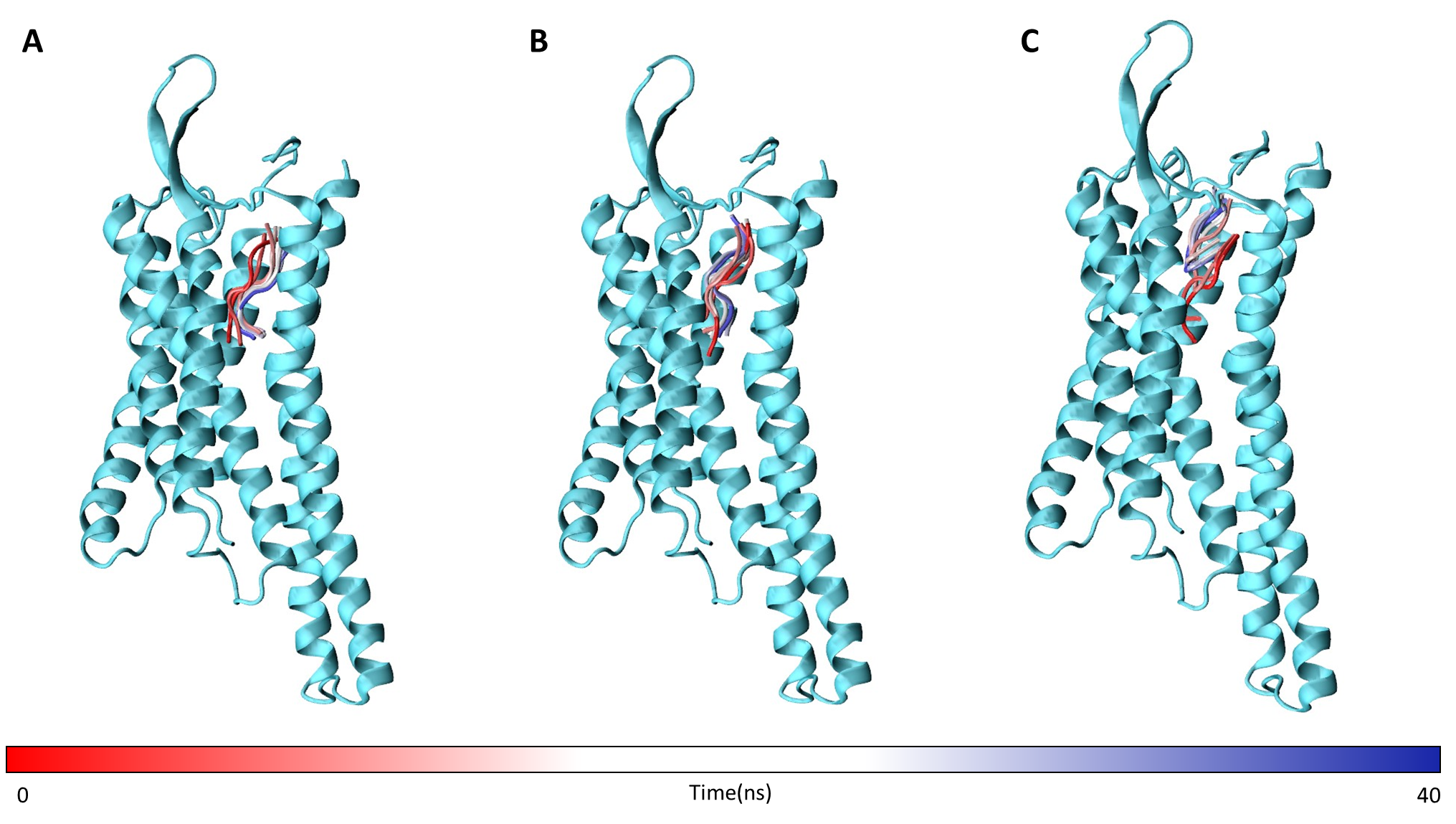


**Figure S2**. Trajectory of GIFGGG (A), GLFGGG (B) and GdIFGGG (C) within GPR120 binding site. The protein is represented by cyan cartoon. The red-to-blue transition reports the stepwise changes of coordinates for each peptide, represented as cartoon, over the 40 ns CMD simulation.
